# Cyclic Electron Flow around Photosystem I Promotes ATP Synthesis Possibly Helping the Rapid Repair of Photodamaged Photosystem II at Low Light

**DOI:** 10.3389/fpls.2018.00239

**Published:** 2018-02-26

**Authors:** Wei Huang, Ying-Jie Yang, Shi-Bao Zhang, Tao Liu

**Affiliations:** ^1^Key Laboratory of Economic Plants and Biotechnology, Kunming Institute of Botany, Chinese Academy of Sciences, Kunming, China; ^2^University of Chinese Academy of Sciences, Beijing, China; ^3^National-Local Joint Engineering Research Center on Germplasm Utilization and Innovation of Chinese Medicinal Materials in Southwest China, Yunnan Agricultural University, Kunming, China

**Keywords:** ATP synthesis, cyclic electron flow, linear electron flow, proton motive force, PSII photoinhibition, P700 oxidation ratio

## Abstract

In higher plants, moderate photoinhibition of photosystem II (PSII) leads to a stimulation of cyclic electron flow (CEF) at low light, which is accompanied by an increase in the P700 oxidation ratio. However, the specific role of CEF stimulation at low light is not well known. Furthermore, the mechanism underlying this increase in P700 oxidation ratio at low light is unclear. To address these questions, intact leaves of the shade-adapted plant *Panax notoginseng* were treated at 2258 μmol photons m^-2^ s^-1^ for 30 min to induce PSII photoinhibition. Before and after this high-light treatment, PSI and PSII activity, the energy quenching in PSII, the redox state of PSI and proton motive force (*pmf*) at a low light of 54 μmol photons m^-2^ s^-1^ were determined at the steady state. After high-light treatment, electron flow through PSII (ETRII) significantly decreased but CEF was remarkably stimulated. The P700 oxidation ratio significantly increased but non-photochemical quenching changed negligibly. Concomitantly, the total *pmf* decreased significantly and the proton gradient (ΔpH) across the thylakoid membrane remained stable. Furthermore, the P700 oxidation ratio was negatively correlated with the value of ETRII. These results suggest that upon PSII photoinhibition, CEF is stimulated to increase the ATP synthesis, facilitating the rapid repair of photodamaged PSII. The increase in P700 oxidation ratio at low light cannot be explained by the change in *pmf*, but is primarily controlled by electron transfer from PSII.

## Introduction

Although light is the driving force of photosynthesis, high light can cause significant photoinhibition of photosystem II (PSII) for leaves of shade-establishing plants (Kitao et al., 2000; Barth et al., 2001; Krause et al., 2004; Huang et al., 2015b, 2016b,c). However, photoinhibited PSII recovers rapidly at low light (He and Chow, 2003; Zhang and Scheller, 2004, Allakhverdiev et al., 2005), due to the fast turnover rate of D1 protein (Aro et al., 1993; Zhang and Scheller, 2004; Allakhverdiev et al., 2005). The rate of PSII repair was reduced upon inhibition of the synthesis of ATP either via PSI or PSII (Allakhverdiev et al., 2005), indicating that the fast repair of photodamaged PSII complexes needs a large amount of ATP in a short time. A previous study indicated that after chilling-induced photoinhibition of PSII, cyclic electron flow (CEF) was significantly stimulated during recovery at low light (Huang et al., 2010). It was proposed that this stimulation of CEF at low light enhanced the synthesis of ATP for the fast repair of PSII (Huang et al., 2010). However, more evidence is needed to clarify the specific role of CEF stimulation at low light after PSII photoinhibition.

During CEF, electrons from either NADPH or ferredoxin are cycled around PSI into the plastoquinone pool. This electron transfer is coupled to proton translocation and generates a proton gradient across thylakoid membranes (ΔpH) (Shikanai and Yamamoto, 2017). In addition to contributing ATP synthesis, another function of ΔpH is the down-regulation of photosynthetic electron transport by acidifying the thylakoid lumen (Shikanai, 2014, 2016). This regulation involves two different mechanisms: one is linked to thermal energy dissipation and dissipates excess absorbed light energy as heat from PSII antennae (Takahashi et al., 2009), and the other one is down-regulation of Cyt *b*_6_/*f* complex activity and controls the rate of electron transfer to PSI (Suorsa et al., 2012, 2016; Tikkanen and Aro, 2014). In angiosperms including *Arabidopsis thaliana*, two pathways of PSI cyclic electron transport are operating (Shikanai, 2007). The first CEF pathway is PGR5-/PGRL1-dependent pathway sensitive to antimycin A (Munekage et al., 2002; Sugimoto et al., 2013), and the other one is dependent on the chloroplast NADH dehydrogenase-like (NDH) complex (Burrows et al., 1998; Shikanai et al., 1998). The contribution of the PGR5-/PGRL1-dependent pathway is more significant in C3 plants. Under high light, the activation of PGR5-/PGRL1-dependent CEF induces the acidification of thylakoid lumen and thus leads to the high levels of P700 oxidation ratio (Suorsa et al., 2012, 2016; Kono et al., 2014; Yamori et al., 2016). By comparison, the P700 oxidation ratio at low light was little affected by the deficiency in PGR5-/PGRL1-dependent CEF (Munekage et al., 2002, 2004; Kono et al., 2014). Upon moderate PSII photoinhibition, the stimulation of CEF at low light was accompanied with an increase in the P700 oxidation ration (Huang et al., 2010). However, it is unclear whether this increased P700 oxidation ratio is caused by the CEF stimulation.

In addition to CEF, electron transfer from PSII (ETRII) plays an important role in affecting the redox state of PSI (Tikkanen et al., 2014; Huang et al., 2016a,c; Suorsa et al., 2016). In *pgr5* plants of *A. thaliana*, severe photoinhibition of PSII is likely to function as the ultimate control of photosynthetic electron transfer allowing the maintenance of P700 optimally oxidized under excess light (Tikkanen et al., 2014). Recently, Suorsa et al. (2016) indicated that *pgr5* plants showed high P700 oxidation ratio under high light when the ETRII was reduced by combining knockout mutations in *PsbO1, PsbP2, PsbQ1, PsbQ2*, and *PsbR* loci. In chilled leaves of tobacco, moderate PSII photoinhibition depressed electron flow to PSI and then increased the P700 oxidation ratio during further chilling treatments (Huang et al., 2016a). Furthermore, chilling-induced photoinhibition of PSII led to a depression of ETRII, which was accompanied with an increase in the P700 oxidation ratio at low light (Huang et al., 2010). We hypothesize that the P700 oxidation ratio at low light is primarily controlled by electron flow from PSII.

In our previous report, we observed that the shade-establishing plant *Panax notoginseng* showed selective photoinhibition of PSII under high-light stress. After short-term exposure to high-light stress, ETRII at low light significantly decreased but CEF was significantly stimulated. Concomitantly, the P700 oxidation ratio largely increased. Our specific objectives were to (1) investigate whether CEF stimulation at low light mainly facilitates the synthesis of ATP; and (2) determine whether the increase in P700 oxidation ratio upon moderate PSII photoinhibition is more related to ETRII rather than the CEF stimulation or the change in *pmf*. In order to address these questions, intact leaves of *Panax notoginseng* were treated at 2258 μmol photons m^-2^ s^-1^ for 30 min. Before and after high-light treatment, the energy distribution in PSII, the redox state of PSI and proton motive force (*pmf*) at a low light of 54 μmol photons m^-2^ s^-1^ were determined.

## Materials and Methods

### Plant Materials and Growth Condition

In the present study, 2-years-old plants of a shade-establishing plant *Panax notoginseng* (Burkill) F. H. Chen ex C. Chow and W. G. Huang were used for experiments. These plants were grown at light condition of 10% sunlight (with maximum mid-day light intensity of approximately 200 μmol photons m^-2^ s^-1^). No water and nutrition stresses were experienced for these plants. 9-weeks-old fully expanded leaves were used for the photosynthetic measurements.

### PSI and PSII Measurements

Photosystem I and PSII parameters were measured at 25°C by simultaneously recorded using a Dual PAM-100 measuring system (Heinz Walz GmbH, Effeltrich, Germany). The chlorophyll fluorescence parameters were calculated as follows: F_v_/F_m_ = (F_m_ - F_o_)/F_m_, Y(II) = ($F_{m}^{\prime }$ - F_s_)/$F_{m}^{\prime }$ (Genty et al., 1989), NPQ = (F_m_ - $F_{m}^{\prime }$)/$F_{m}^{\prime }$. *F*_o_ and *F*_m_ are the minimum and maximum fluorescence after dark acclimation, respectively. *F*_s_ is the light-adapted steady-state fluorescence. *F*_o_ and *F*_m_ were determined after dark acclimation for 30 min before and after high-light treatment. The PSI photosynthetic parameters were measured by Dual PAM-100 based on P700 signal (difference of intensities of 830 and 875 nm pulse-modulated measuring light reaching the photodetector) (Klughammer and Schreiber, 2008). The P700^+^ signals (*P*) may vary between a minimal (P700 fully reduced) and a maximal level (P700 fully oxidized). The maximum level (*P*_m_) was determined with application of a saturation pulse (600 ms and 10000 μmol photons m^-2^ s^-1^) after pre-illumination with far-red light, and *P*_m_ was used to estimate the PSI activity. *P*_m_′ was determined similar to *P*_m_ but with actinic light instead of far-red light. The quantum yield of PSI was calculated as Y (I) = ($P_{m}^{\prime }$- P)/P_m_. The P700 oxidation ratio in a given actinic light was calculated as Y (ND) = P/P_m_. The quantum yield of PSI non-photochemical energy dissipation due to acceptor-side limitation was calculated as Y (NA) = (P_m_ - $P_{m}^{\prime }$)/P_m_. The steady state values for Y(II), NPQ, Y(I), and Y(ND) were measured after light acclimation at low light for 20 min.

Photosynthetic electron flow through PSI and PSII were calculated as: ETRII = Y (II) ×PPFD ×0.84 ×0.5 (Krall and Edwards, 1992), ETRI = Y (I) ×PPFD×0.84 ×0.5 (Yamori et al., 2011), where 0.5 is assumed to be the proportion of absorbed light reaching PSI or PSII, and 0.84 is assumed to be the absorptance (the fraction of the incident light absorbed by leaves). The extent of CEF activation was estimated as ETRI/ETRII ratio (Yamori et al., 2011, 2015). It should be noted that the 1:1 excitation partitioning between PSI and PSII may be not real, because shade plants tend to increase the PSII/PSI ratio (Lunde et al., 2003; Tikkanen et al., 2006; Grieco et al., 2012). Furthermore, in this study, PSI and PSII parameters were measured using red actinic light (635 nm) provided by Dual-PAM 100 (Walz, Germany). Because red actinic light favors the excitation of PSII over that of PSI, the excitation energy distribution from LHCII to PSII and PSI may be affected by the red actinic light (Tikkanen et al., 2017).

### Electrochromic Shift Analysis

The ECS signal was monitored as the absorbance change at 515 nm using a Dual-PAM-100 (Walz, Effeltrich, Germany) equipped with a P515/535 emitter-detector module (Walz). The ECS signal was obtained after 20 min of illumination at 54 μmol photons m^-2^ s^-1^, afterwards, the ECS decay was measured by switching off the actinic light for 30 s. The analysis of ECS dark interval relaxation kinetics (DIRK_ECS_) was done by the method of Sacksteder et al. (2001) and Takizawa et al. (2008). Total *pmf* was estimated from the total amplitude of the rapid decay of the ECS signal during 300 ms dark interval. The slow relaxation of ECS signal enabled to recognize the contribution of proton gradient across the thylakoid membranes (ΔpH). The time constant of the first-order ECS relaxation (τ_ECS_) is inversely proportional to the proton conductivity (*g*_H_^+^) of the thylakoid membrane through the ATP synthase (Sacksteder and Kramer, 2000; Cruz et al., 2005). As a result, *g*_H_^+^ was estimated as the inverse of the decay time constant [1/τ_ECS_].

### Photoinhibitory Treatments

After dark acclimation for 30 min, F_v_/F_m_ and *P*_m_ were measured in intact leaves. Afterwards, these intact leaves were light-adapted at 59 μmol photons m^-2^ s^-1^ for 20 min and parameters of chlorophyll fluorescence, P700 signal and ECS signal were recorded. Then the actinic light was changed to 2258 μmol photons m^-2^ s^-1^. After exposure to this high light for 30 min, the actinic light was changed to 59 μmol photons m^-2^ s^-1^ immediately and the photosynthetic parameters were recorded after light acclimation for 20 min. Finally, F_v_/F_m_ and *P*_m_ were measured after dark acclimation for 30 min.

### Statistical Analysis

The results were displayed as mean values of five independent experiments. Independent *T*-test was used at α = 0.05 significance level to determine whether significant differences existed between different treatments. All statistical analyses were performed using SPSS 16.0.

## Results

**FIGURE 1:**
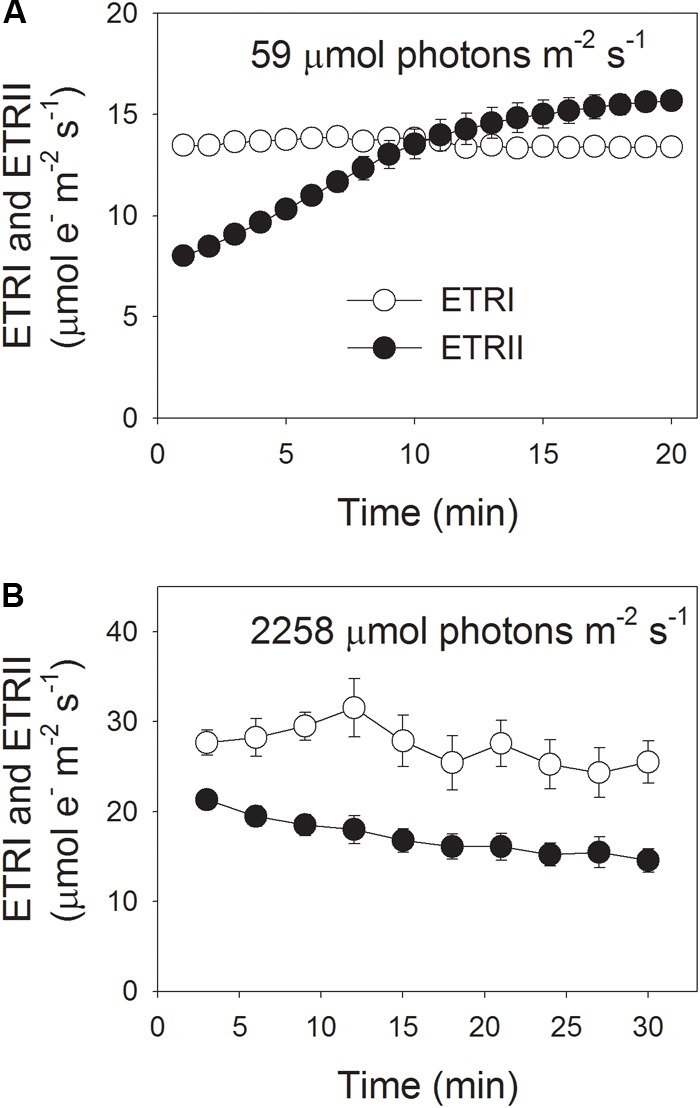
Photosynthetic electron flow at low light **(A)** and high light **(B)**. **(A)** Dark-acclimated leaves were exposed to a low light of 59 μmol photons m^-2^ s^-1^ for 20 min. **(B)** After illumination at 59 μmol photons m^-2^ s^-1^ for 20 min to activate the electron sink in photosynthesis, leaves were exposed to a high light of 2258 μmol photons m^-2^ s^-1^ for 30 min. Values are means ± SE (*n* = 5).

**FIGURE 2:**
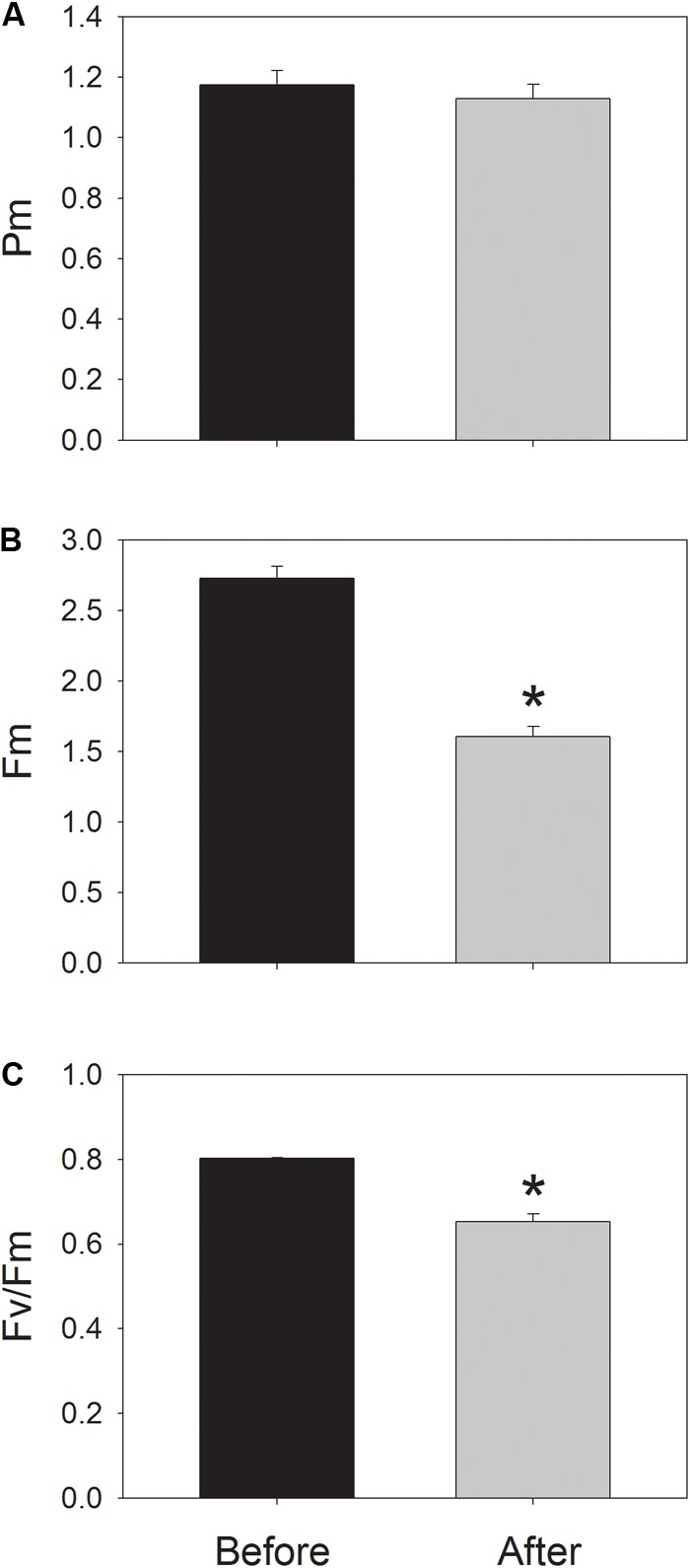
Values for the maximum photo-oxidizable P700 (*P*_m_) **(A)**, the maximum fluorescence intensity (*F*_m_) **(B)**, and the maximum quantum yield of PSII (F_v_/F_m_) **(C)** before and after treatment at 2258 μmol photons m^-2^ s^-1^ for 30 min. *P*_m_ and *F*_v_/*F*_m_ were measured after dark-acclimation for 30 min. Values are means ± SE (*n* = 5). Asterisk indicates a significant (*P* < 0.05) change after the high-light treatment.

During photosynthetic induction at 59 μmol photons m^-2^ s^-1^, ETRII gradually increased and reached a steady state at about 18 min (**Figure 1A**). By comparison, ETRI was constant during this induction phase (**Figure 1A**). After onset of this low light, ETRI was much higher than ETRII. However, after a 20-min photosynthetic induction, ETRI was lower than ETRII. These results suggested the activation of CEF during the initial phase of induction, in accordance with previous studies (Joliot and Joliot, 2002, 2005; Makino et al., 2002). After this photosynthetic induction at low light, leaves were illuminated at a high light of 2258 μmol photons m^-2^ s^-1^ for 30 min. Interestingly, ETRII gradually decreased during the high-light treatment (**Figure 1B**). Meanwhile, the value of ETRI was higher than that of ETRII (**Figure 1B**), indicating the activation of CEF under high light. After this high-light treatment, the maximum photo-oxidizable P700 (*P*_m_) was maintained stable (**Figure 2A**). By comparison, the maximum fluorescence intensity (*F*_m_) decreased by 40% and the maximum quantum yield of PSII (F_v_/F_m_) decreased from 0.80 to 0.65 (**Figures 2B,C**). These results indicated the selective photoinhibition of PSII in leaves of *Panax notoginseng* under high-light stress.

**FIGURE 3:**
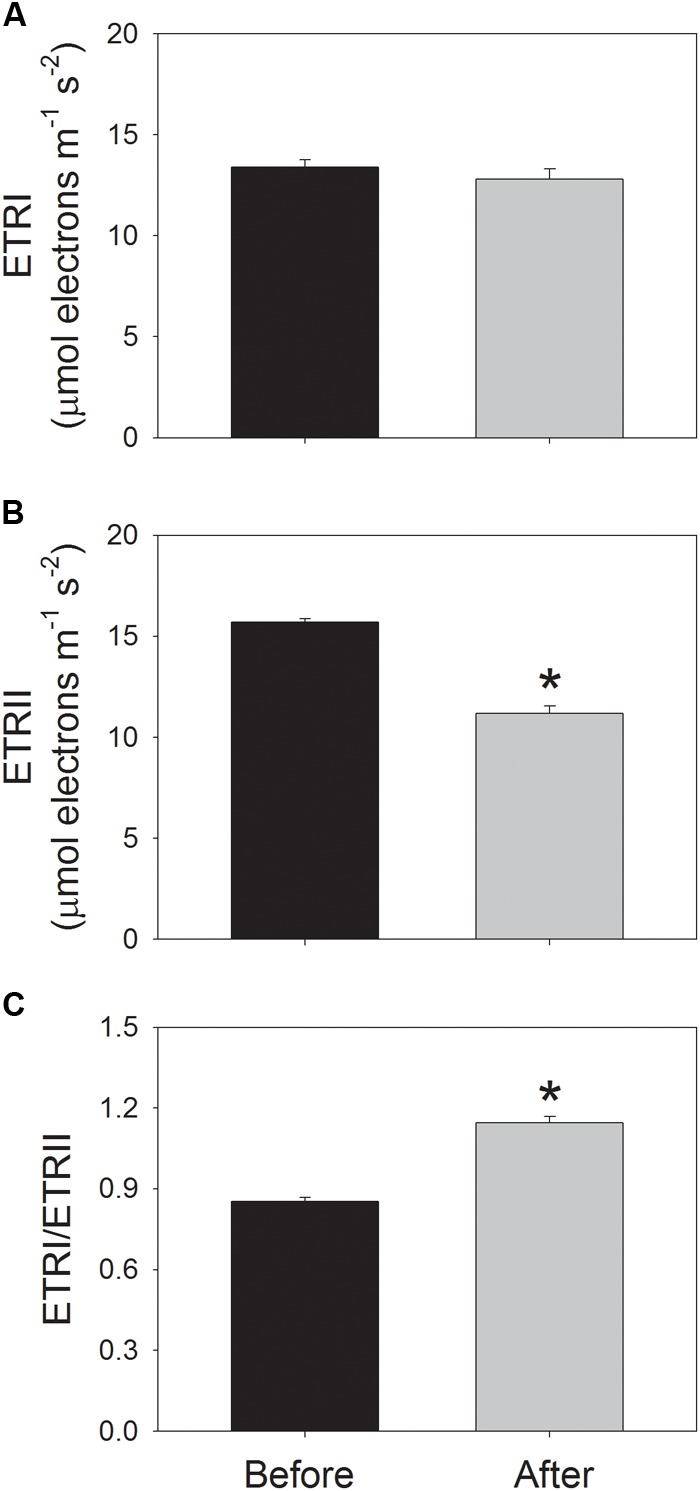
Values for the electron flow through PSI (ETRI) **(A)**, the electron flow from PSII (ETRII) **(B)**, and the ETRI/ETRII ratio **(C)** at low light. Before and after high-light treatment, ETRI and ETRII were measured after illumination at 59 μmol photons m^-2^ s^-1^ for 20 min. Values are means ± SE (*n* = 5). Asterisk indicates a significant (*P* < 0.05) change after the high-light treatment.

Before high-light treatment, mature leaves were illuminated at a low light of 59 μmol photons m^-2^ s^-1^ for 20 min to activate photosynthesis, and then values for ETRI, ETRII, Y(ND), and NPQ were recorded. After high-light treatment for 30 min, values for ETRI, ETRII, Y(ND), and NPQ were recorded following a new acclimation phase of 20 min to low light. Before high-light treatment, values for ETRI and ETRII at 59 μmol photons m^-2^ s^-1^ were 13.4 and 15.7 μmol electrons m^-2^ s^-1^, respectively (**Figures 3A,B**). After high-light treatment, ETRI and ETRII at low light were 12.8 and 11.2 μmol electrons m^-2^ s^-1^, respectively (**Figures 3A,B**). The value of ETRII at 59 μmol photons m^-2^ s^-1^ decreased by approximately 30% after photoinhibitory treatment, indicating the depression of ETRII upon moderate PSII photoinhibition. Before high-light treatment, the value of ETRI/ETRII ratio at 59 μmol photons m^-2^ s^-1^ was 0.85 (**Figure 3C**). After photoinhibitory treatment, the ETRI/ETRII ratio significantly increased to 1.14 (**Figure 3C**). These results suggested the stimulation of CEF at low light upon PSII photoinhibition (Yamori et al., 2011, 2015).

**FIGURE 4:**
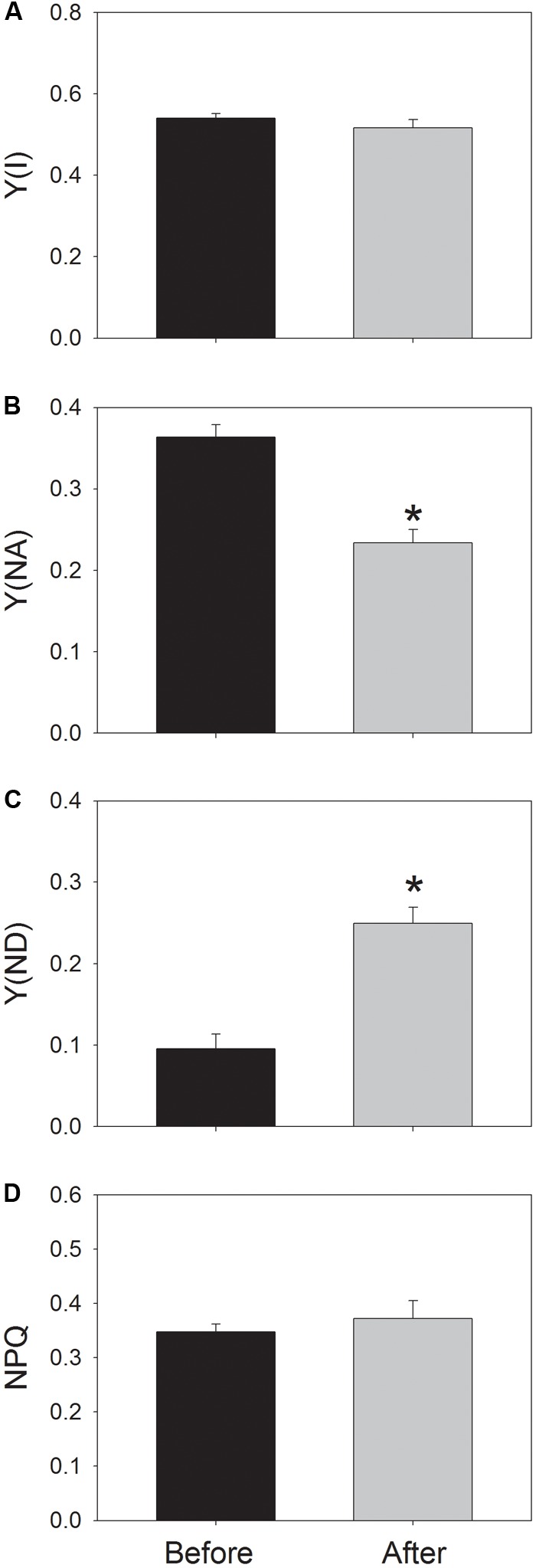
Values for Y(I) **(A)**, Y(NA) **(B)**, Y(ND) **(C)**, and NPQ **(D)** at low light. Before and after high-light treatment, Y(ND) and NPQ were measured after illumination at 59 μmol photons m^-2^ s^-1^ for 20 min. Values are means ± SE (*n* = 5). Asterisk indicates a significant (*P* < 0.05) change after high-light treatment.

After the high-light treatment, the steady-state value of Y(I) at 59 μmol photons m^-2^ s^-1^ did not change (**Figure 4A**). Interestingly, Y(NA) significantly decreased from 0.36 to 0.23 (**Figure 4B**), and Y(ND) significantly increased from 0.1 to 0.25 (**Figure 4C**). These results indicated the change in redox state of PSI at low light after photoinhibitory treatment. Meanwhile, the value of NPQ changed slightly (**Figure 4D**). As a result, the moderate PSII photoinhibition had different effects on P700 oxidation ratio and NPQ at low light.

**FIGURE 5:**
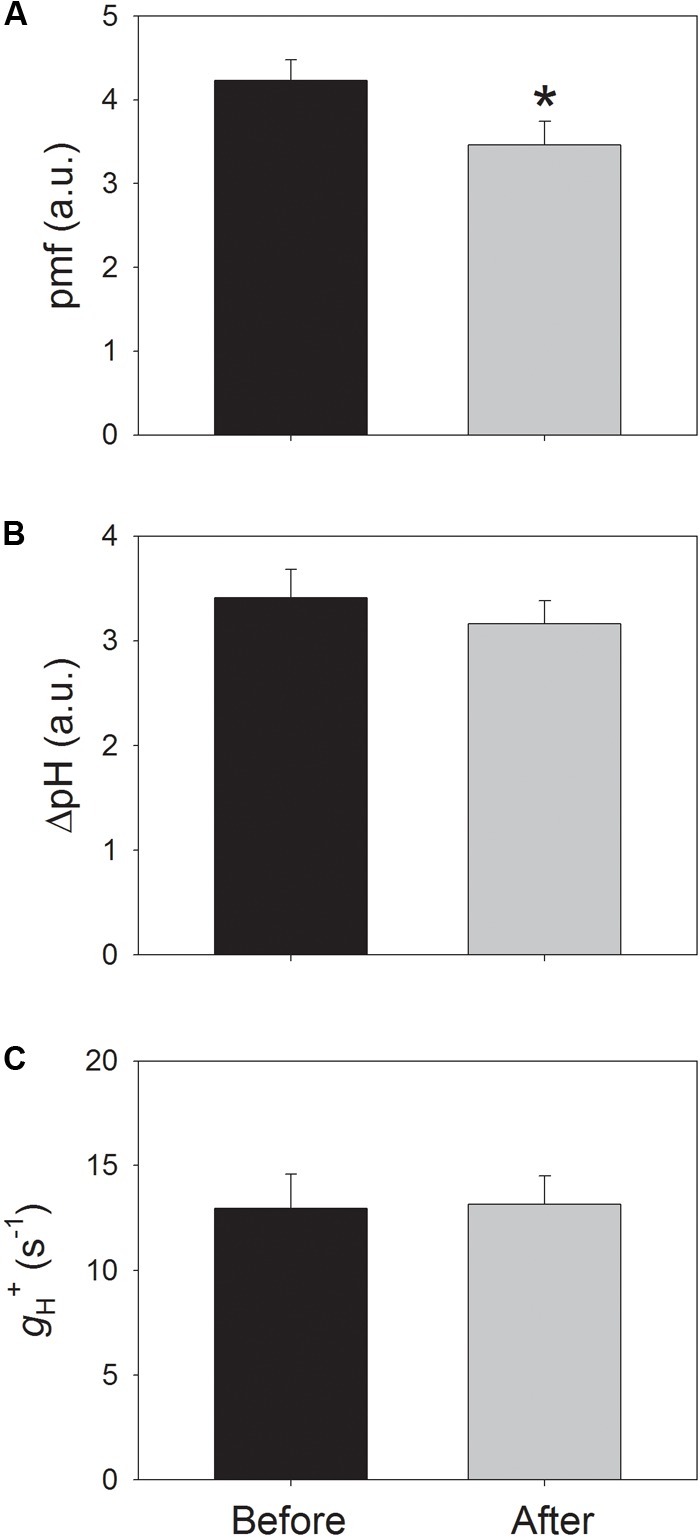
Values for the total proton motive force (*pmf*) **(A)**, the proton gradient (ΔpH) across the thylakoid membranes **(B)**, and the proton conductivity (*g*_H_^+^) of the thylakoid membrane **(C)** at low light. All parameters were measured after adaptation at 54 μmol photons m^-2^ s^-1^ for 20 min. Values are means ± SE (*n* = 5). Asterisk indicates a significant (*P* < 0.05) change after high-light treatment.

In order to clarify whether the increase in Y(ND) is caused by an increase in *pmf*, the electrochromic shift signals at 54 μmol photons m^-2^ s^-1^ were determined after light acclimation for 20 min before and after the high-light treatment. Interestingly, the total *pmf* significantly decreased by 18% after high-light treatment (**Figure 5A**), but the ΔpH level changed insignificantly (**Figure 5B**). Because the formation of *pmf* can be affected by the thylakoid proton conductivity, the proton conductivity (*g*_H_^+^) of the thylakoid membrane at this low light was also examined before and after high-light treatment. The result showed that *g*_H_^+^ did not change with the high-light treatment (**Figure 5C**), suggesting that the high-light treatment hardly affected the activity of the chloroplast ATP synthase at low light. Because the decrease of 30% in ETRII was accompanied by a decrease of 18% in *pmf*, the stimulation of CEF at low light partly compensated for the formation of *pmf* and ΔpH.

## Discussion

### The Role of CEF Stimulation at Low Light

It has been indicated that CEF plays important roles in sustaining photosynthesis and plant growth (Yamori and Shikanai, 2016). Under high light, CEF is now thought to be essential for balancing the ATP/NADPH energy budget as well as for protecting PSI and PSII from damage (Munekage et al., 2002, 2004; Takahashi et al., 2009; Suorsa et al., 2012, 2016; Walker et al., 2014; Huang et al., 2015a, 2017b). At low light intensity, CEF plays an important role in optimizing photosynthetic CO_2_ assimilation probably via the supply of extra ATP (Yamori et al., 2011, 2015; Nishikawa et al., 2012; Huang et al., 2015a). The main role of CEF is modulated flexibly in response to light intensity. In particular, CEF mainly contributes to balancing the ATP/NADPH energy budget under sub-saturating light intensities, but mainly protects photosynthetic apparatus against photoinhibition when exposed to saturating light intensities via acidification of the thylakoid lumen (Huang et al., 2015a). Interestingly, Huang et al. (2010) found that CEF was significantly stimulated at low light after chilling-induced photoinhibition of PSII and hypothesized that this CEF stimulation mainly enhanced the synthesis of ATP for the fast repair of PSII. However, more evidence was needed to support this hypothesis. In the present study, we observed that the value of ETRI/ETRII at low light significantly increased (**Figure 3C**), indicating the stimulation of CEF around PSI (Huang et al., 2011, 2012, 2017a,b; Yamori et al., 2011, 2015). Concomitantly, the amplitude of *pmf* decreased significantly and ΔpH declined slightly. These results indicated that the stimulation of CEF did not trigger the ΔpH-dependent down-regulation of photosynthetic electron transport. In the other words, this stimulation of CEF at low light mainly facilitated the synthesis of ATP.

Although the proton conductivity of the chloroplast ATP synthase was not affected by the high-light treatment, the smaller size of the *pmf* resulted in a decrease in the actual rate of ATP synthesis. The rates of PSII repair and photosynthetic CO_2_ assimilation at low light are mainly limited by the rate of ATP synthesis (Allakhverdiev et al., 2005; Yamori et al., 2011, 2015; Nishikawa et al., 2012). Because the ETRII significantly declined upon PSII photoinhibition, the rate of ATP synthesis via ETRII was remarkably reduced. In order to balance the ATP/NADPH ratio required by primary metabolism and the fast repair of PSII, a flexible mechanism is needed to provide extra ATP. Under this condition, ETRII decreased by 30% but the total *pmf* decreased by just 18%. These results indicate that the stimulation of CEF compensated for the reduction in ETRII-dependent formation of *pmf*, enhancing the synthesis of ATP. The rapid repair of photodamaged PSII is dependent on ATP synthesis (Allakhverdiev et al., 2005). Acceleration of CEF around PSI increased the intracellular concentration of ATP, thus accelerating the rate of PSII repair in *Synechocystis* (Allakhverdiev et al., 2005). Taken together, we propose that the stimulation of CEF at low light plays an important role in the fast repair of PSII activity via an additional ATP synthesis.

### Change in P700 Oxidation Ratio at Low Light Is Not Related to *pmf*

An interesting phenomenon is that the P700 oxidation ratio at low light significantly increased upon moderate PSII photoinhibition (Huang et al., 2010), which is also showed in the present study. Under conditions in which absorbed light is in excess of the requirements for photosynthesis, CEF-dependent generation of ΔpH down-regulates the activity of Cyt *b*_6_/*f* complex activity and controls electron flow from PSII to PSI (Suorsa et al., 2012, 2016; Shikanai, 2014, 2016), optimizing the redox state of P700 in PSI and minimizing ROS production in PSI during photosynthesis. In *pgr5*-plants of *A. thaliana*, P700 is reduced by electrons in the light due to the loss of *pmf* generation. However, in the *pgr5* mutant accumulating flavodiiron protein, the level of *pmf* was also restored to the wild-type level, and thus P700 was oxidized as in the wild-type (Yamamoto et al., 2016). In chlorophyll *b*-deficient wheat mutant lines, insufficient thylakoid proton gradient leads to over-reduction of PSI acceptor side and thus PSI photoinhibition under high light or high temperature (Brestic et al., 2015, 2016). Recently, some studies reported that the increased activity of the chloroplast ATP synthase impaired the formation of *pmf* and caused the over-reduction of photosynthetic electron transport chain, resulting in photodamage of PSI under high light and fluctuating light (Kanazawa et al., 2017; Takagi et al., 2017). These reports support the critical role of *pmf* in maintaining P700 optimally oxidized under excess light energy.

Our present results indicated that after the high-light treatment for 30 min, the total *pmf* formed at this low light significantly declined and the formation of ΔpH changed slightly (**Figures 5A,B**). Meanwhile, the P700 oxidation ratio significantly increased from 0.1 (before treatment) to 0.25 (after treatment) (**Figure 4C**). These results strongly indicate that this increase in P700 oxidation ratio cannot be explained by the change in *pmf*, which is largely different from the correlation between Y(ND) and *pmf* under high light (Yamamoto et al., 2016; Takagi et al., 2017). Therefore, the regulating effect of *pmf* on P700 oxidation ratio is minimal at low light but becomes particularly crucial under high light or fluctuating light. Under high light, a high value of Y(ND) is usually accompanied with a high value of NPQ (Munekage et al., 2002, 2004; Kono et al., 2014; Zivcak et al., 2015), due to the increase in *pmf*. The impairment of ΔpH formation leads to decreased levels of Y(ND) and NPQ under high light (Suorsa et al., 2012; Kono et al., 2014; Yamamoto et al., 2016; Kanazawa et al., 2017). The induction of NPQ at low light is mainly determined by the level of lumen acidification. After high-light treatment, the extent of lumen acidification did not change (**Figure 5B**), and NPQ remained stable (**Figure 4D**). By comparison, Y(ND) significantly increased. These results indicate that at low light, Y(ND) and NPQ were controlled by different regulatory mechanisms.

### ETRII Controls P700 Oxidation Ratio at Low Light

PSI becomes over-reduced only when electron flow from PSII exceeds the capacity of PSI electron acceptors to cope with the electrons. When electron flow to PSI is limited, PSI is extremely tolerant against light stress in the *pgr5* plants of *A. thaliana* (Suorsa et al., 2012, 2016; Tikkanen et al., 2014). For the shade-establishing plant *Psychotria rubra*, PSI activity was insusceptible to high-light stress in the presence of DCMU (Huang et al., 2016c). In chilled leaves of tobacco, moderate PSII photoinhibition allowed the maintenance of P700 optimally oxidized and then protected PSI activity against further photodamage (Huang et al., 2016a). These results indicated the important role of ETRII in controlling the redox state of PSI under high light or chilling-light stresses. Our present results showed that the moderate PSII photoinhibition led to a significant depression of ETRII at low light. Meanwhile, the PSI activity and the activity of the chloroplast ATP synthase changed slightly, and the total *pmf* and ΔpH did not increase. As a result, the reduction in ETRII was not caused by the ΔpH-dependent photosynthetic control via Cyt *b*_6_/*f* complex, but was probably induced by the decrease in PSII activity (Tikkanen et al., 2014). The decreased supply of electrons from PSII to PSI led to the increased level of P700 oxidation. Thus, the P700 oxidation ratio at low light was largely controlled by ETRII.

## Conclusion

We found that the selective photoinhibition of PSII induced a stimulation of CEF and an increase in P700 oxidation ratio at low light. The stimulation of CEF did not trigger the ΔpH-dependent down-regulation of photosynthetic electron transport. As a result, this stimulation of CEF at low light mainly facilitated the synthesis of ATP, which is essential for the rapid repair of photodamaged PSII. The increase in P700 oxidation ratio could not be explained by the change in ΔpH-dependent photosynthetic control at the Cyt *b*_6_/*f* complex, but was primarily caused by the decreased supply of electrons from PSII to PSI.

## Author Contributions

WH, S-BZ, and TL conceived and designed the research. WH and Y-JY conducted the experiments. WH, Y-JY, S-BZ, and TL analyzed the data. WH wrote the first draft of the manuscript, which was intensively edited by all the authors.

## Conflict of Interest Statement

The authors declare that the research was conducted in the absence of any commercial or financial relationships that could be construed as a potential conflict of interest.
